# Antibacterial, cytotoxic and genotoxic activity of nitrogenated and haloid derivatives of C_50_–C_60_ and C_70_–C_120_ polyprenol homologs

**DOI:** 10.1186/s12944-016-0345-x

**Published:** 2016-10-10

**Authors:** Ran Tao, Cheng-zhang Wang, Jian-zhong Ye, Hao Zhou, Hong-xia Chen, Chang-wei Zhang

**Affiliations:** 1 Institute of Chemical Industry of Forest Products, CAF, Nanjing, Jiangsu Province 210042 China; 2 Research Institute of Forestry New Technology, CAF, Beijing, 100091 China

**Keywords:** Polyprenol, Nitrogenated derivatives, Haloid derivatives, Antibacterial activity, Cytotoxic activity, Genotoxic activity

## Abstract

**Background:**

Polyprenol is an important lipid with many bioactive effects. The study on differences in bioactive effects of polyprenol derivatives having different isoprene units are seldom reported and it is helpful to find out which type of polyprenol derivatives are effective for treating A549/HepG2 cells and *E. coli* /*S. aureus*.

**Methods:**

All tested polyprenol derivatives were measured with inhibition halos by Oxford cup assays. MIC values were assessed by the broth dilution method. Time-killing curve studies were conducted in duplicate on separate days. Cytotoxicity study was measured by the MTT assay and genotoxic study was evaluated by comet assay.

**Results:**

With regard to antibacterial activity, the sensitivities to the quaternary polyprenyl ammonium salt derivatives GAS and MAS were 31.3 μg/mL and 15.6–31.3 μg/mL, respectively. GAS and MAS exhibited cytotoxic activity toward HepG2 cells (IC_50_ of 10.1–11.6 μg/mL), which was stronger than that exhibited toward A549 cells (IC_50_ of 13.8–13.9 μg/mL). The bactericidal activity of MAS was stronger than that of GAS at the same concentration at least 48 h. The DNA damage in A549 and HepG2 cells exposed to all 10, 20 and 40 μg/mL MAS was statistically significant in comparison to the control. Our results indicate a dose-dependent increment in DNA damage in A549 and HepG2 cells exposed to 10, 20 and 40 μg/mL MAS for both the percentage of DNA in the tail and tail moment.

**Conclusion:**

The quaternary ammonium salt derivatives GAS and MAS exhibited higher antibacterial (*E. coli* and *S. aureus*) and cytotoxic activity (A549 and HepG2 cells) than the other derivatives evaluated in this study. The DNA damage in HepG2 cells suggests that MAS induced A549 and HepG2 cells death via apoptotic pathway. Our results provide new evidence supporting the medical use of polyprenol derivatives against bacterial and tumor diseases.

## Background

*Ginkgo biloba* (Ginkgoopsida, Ginkgoales, Ginkgoaceae, *Ginkgo biloba*L.) leaf polyprenol (GBP) and *Morus alba* (Dicotyledoneae, Rosales, Moraceae, *Morus* L.) leaf polyprenol (MBP) are long-chain polyisoprenoid alcohols. These molecules are typically composed of a number of unsaturated isoprene units; they are a type of betulaprenol with an *E*, *E*-farnesyl residue at the *ω*-end of the prenyl chain and are terminated by an isoprene unit bearing a primary hydroxyl group. GBP generally consists of 70 to 120 carbons [1], and MBP consists of 50 to 60 carbons [2]. Polyprenols have various medical functions, such as antiviral [3], antitumor [4], hepatoprotective [5], anti-Alzheimer [6] and synergistic antibacterial [1] activities. Indeed, the bioactivity of polyprenols toward single-celled organisms and the cells of higher organisms in vitro has gradually become a focus of lipid research. For example, one study determined that alloprenols, a newly discovered class of polyprenols in plants that contain an *α*-isoprene unit in the trans-(*E*)-configuration, increase membrane permeability to a greater extent than polyprenols with the more common *cis*-configuration [7]. Further evidence suggests that an increase in dolichol length can slightly enhance membrane destabilization at earlier times [8]. Good antibacterial activity against both gram-negative and gram-positive bacteria (MIC 6.25–25.0 mg/mL and MBC 12.50–25.0 mg/mL) has been demonstrated in vitro by polyprenols isolated from the leaves of *Kirganelia reticulate* [9], and we previously reported MIC values of 31.3 μg/mL and MBC and MFC values of 125 μg/mL for GBP (inhibition halos 13.4–13.8 mm) [1]. Thus, it can be inferred that different polyprenols possess different antibacterial activities. Moreover, different polyprenol derivatives also show different antibacterial activities. In fact, our recent study of five GBP derivatives—polyprenyl acetate (GPA), n-amyl polypentylene (GAP), polyprenyl-3-isoamyl benzyl ether (GPB), polyprenyl dichlorophosphite (GPD) and polyprenyl phosphate disodium salt (GPP) - synthesized from GBPs (C_70_–C_120_) suggested a decrease in the antibacterial/antifungal activities and synergistic effect of alkyl-substituted GBP derivatives; however, an increase was observed with a phosphate-substituted GBP derivative [10]. As phosphorylated polyprenols generally possess higher activity than free polyprenols, studying polyprenyl and dolichyl phosphate analogs modified at the anionic group is of substantial interest, particularly with regard to comparisons of their biological activities [11].

Previously, it has been reported that amino derivatives of linear isoprenoids possess immunomodulating, antiulcer, and antithrombotic properties [12], and prenylamines demonstrate anti-vaccinia virus activity as well as human interferon-inducing properties [13]. Veselovsky [14] developed a simple method for synthesizing polyprenyl amines of polyprenols from mulberry leaves and pine needles via a two-step transformation. Sizova^11^ synthesized a quaternary polyprenyl ammonium salt using mulberry leaf polyprenols with triethylamine in the presence of phosphorus oxychloride, and cationic polyprenyl derivatives may be useful for transfection and immunological studies. In the present study, a microemulsion containing these nitrogenated and haloid polyprenol derivatives was investigated and found to have antibacterial, cytotoxic and genotoxic properties. The compounds tested are polyprenyl phthalimide derivatives (GPH from GBP and MPH from MBP), polyprenyl amine derivatives (GAM from GBP and MAM from MBP), quaternary polyprenyl ammonium salt derivatives (GAS from GBP and MAS from MBP), polyprenyl trifluoroacetate derivatives (GTF from GBP and MTF from MBP) and polyprenyl chloroacetate derivatives (GCH from GBP and MCH from MBP) of polyprenols of two different chain lengths isolated from the leaves of *Ginkgo biloba* (GBL) and *Morus alba* (MBL). The study on differences in bioactive effects among polyprenol derivatives having different isoprene units is seldom reported and it could be helpful to find out which type of polyprenol derivatives are effective for treating A549/HepG2 cells and *E. coli*/*S. aureus. E. coli* (Gram-negative bacteria) and *S. aureus* (Gram positive bacteria) are the two most common and typical kinds of pathogenic bacteria. The study on the antibacterial activities against *E. coli* and *S. aureus* is representative and significant to treat bacterial contamination and infection. Lung and liver cancer are two types of cancer having a higher incidence in developing countries. A549 (from lung cancer cell) and HepG2 (from liver cancer cell) cells are good objects for studying on treating lung and liver cancer cells. Antibacterial, cytotoxic and genotoxic activities of these derivatives of C_50_–C_60_ and C_70_–C_120_ polyprenols have seldom been reported to date.

## Results and discussion

### Structural determination

**Fig. 1:**
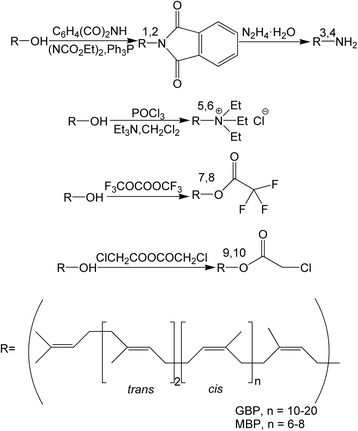
Synthesis of the compounds. 1.GPH 2.MPH 3.GAM 4.MAM 5.GAS 6.MAS 7.GTF 8.MTF 9.GCH 10.MCH

The structures (Fig. 1) of GPH, MPH, GAM, MAM, GAS, MAS, GTF, MTF, GCH and MCH were determined by ^1^H-NMR data reported in this paper and the corresponding references [14].

### Antibacterial activity

The antibacterial activities of GPH, MPH, GAM, MAM, GAS, MAS, GTF, MTF, GCH and MCH against *E. coli* and *S. aureus* were assessed at 500 μg/mL, and their potency was quantitatively assessed based on inhibition halos (Table 1) and MIC values (Table 2). Analysis of variance (Tukey’s test at 5 % probability) indicated a significant difference (*p* < 0.05) among all the samples. Among the samples tested, the MIC values indicated that the polyprenyl ammonium salt derivatives GAS and MAS had the highest activity and inhibited the growth of both bacteria. The MIC values for GAS and MAS were 31.3 μg/mL and in the range of 15.6–31.3 μg/mL, respectively. The inhibition halo diameters from large to small were MAS, GAS, GTF ≈ MAM, GAM ≈ MTF, GCH ≈ GPH ≈ MCH, MPH for *E. coli* and MAS, GAS, GTF, MAM ≈ MTF, GAM ≈ GCH, MCH ≈ GPH, MPH for *S. aureus*. Most importantly, all samples were effective at inhibiting the growth of the two examined bacteria. Moreover, the same types of derivatives showed different antibacterial activities, with those of the phthalimides, trifluoroacetate derivatives of GBP and chloroacetate derivatives of GBP being higher than those of MBP. Overall, the antibacterial activities of the amine and quaternary ammonium salt derivatives of GBP were lower than those of MBP. These results suggest that the quaternary polyprenyl ammonium salt derivatives (GAS and MAS) have higher antibacterial activity (*E. coli* and *S. aureus*) compared with the other nitrogenated and haloid polyprenol derivatives. We therefore next decided to examine time-killing curves for GAS and MAS at different concentration.

**Table 1:** Comparison of the inhibition halos among different samples (Tukey’s test at 5 % probability)

Samples	Diameter of the inhibition halos (mm) ± SEM, *n* = 3	
	*E. coli*	*S. aureus*
GPH	15.6 ± 0.3 a	16.0 ± 0.2 a
MPH	15.2 ± 0.3 b	15.1 ± 0.2 b
GAM	16.4 ± 0.3 c	16.8 ± 0.2 c
MAM	16.9 ± 0.3 d	17.2 ± 0.2 d
GAS	19.1 ± 0.3 e	19.8 ± 0.2 e
MAS	19.8 ± 0.3 f	20.9 ± 0.2 f
GTF	16.9 ± 0.3 d	17.5 ± 0.2 g
MTF	16.3 ± 0.3 c	17.1 ± 0.2 d
GCH	15.7 ± 0.3 a	16.7 ± 0.2 c
MCH	15.6 ± 0.3 a	16.1 ± 0.2 a
GS	24.1 ± 0.2	23.0 ± 0.3

The same lowercase letters in the column of the same group indicate no statistical difference (Tukey’s HSD test, *p* > 0.05). *GS* Gentamycin sulfate

**Table 2:** Minimum inhibitory concentration (μg/mL) of different samples

Samples	MIC values (μg/mL)	
	*E. coli*	*S. aureus*
GPH	125	125
MPH	125	125
GAM	62.5	62.5
MAM	62.5	62.5
GAS	31.3	31.3
MAS	31.3	15.6
GTF	62.5	62.5
MTF	62.5	62.5
GCH	125	125
MCH	125	62.5
GS	7.8	7.8

### Time-killing curves of *E. coli* and *S. aureus* in GAS and MAS at different concentration

**Fig. 2:**
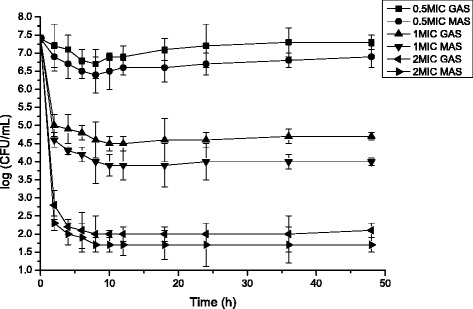
Time-killing curves for *Escherichia coli* treated with GAS and MAS at different concentration (Mean ± SD, *n* = 3)

**Fig. 3:**
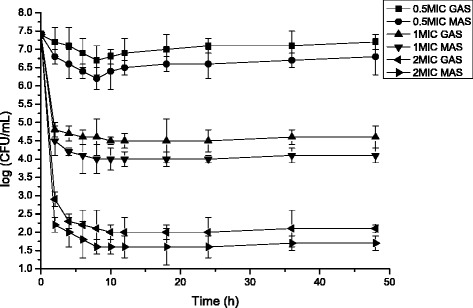
Time-killing curves for *Staphylococcus aureus* treated with GAS and MAS at different concentration (Mean ± SD, *n* = 3)

The time-killing curves for *E. coli* and *S. aureus* treated with GAS and MAS at different concentration (0.5, 1 and 2 times the MIC) are shown in Figs. 2 and 3. Overall, the bactericidal activity of MAS was stronger (Tukey’s test, p < 0.05) than that of GAS at the same concentration at least 48 h (we did not test longer durations). 2 times the MIC concentration of GAS and MAS treatment resulted in a rapid decline (decreasing at least 99.9 %, ≥ 3 × log) of initial population of *E. coli* and *S. aureus* within 2 h. By contrast, 1 times the MIC concentration of GAS and MAS were able to decrease 99.0 % (≥2 × log) of *E. coli* and *S. aureus* population within 2 h. 0.5 and 2 times the MIC of GAS and MAS were strongest antibacterial effect until them were treated for 8 h while 1 times the MIC of GAS and MAS were strongest antibacterial effect until them were treated for 10 h. 0.5 times the MIC of GAS were inactivated against *E. coli* and *S. aureus* after 24 h.

An increasing number of studies have shown that polymeric quaternary ammonium compounds exhibit greater bactericidal activities than their precursor monomers, which may be due by the antibacterial action of quaternary ammonium salts [15, 16]. There are four basic key factors in the antibacterial activity of polymeric quaternary ammonium salts [17]: (1) adsorption onto the negatively charged bacterial cell surface; (2) penetration of the cell wall; (3) binding to the plasma membrane; and (4) damaging the plasma membrane. It is considered that similar to the addition of polyprenyl groups, the polymerization of monomers could increase the positive charge density of the polymer coil, which could further enhance the ability of a compound to efficiently adsorb onto the negatively charged bacterial surface. Therefore, increasing the number of polyprenyl groups could help promote this process (1). Many researchers have noted that the antibacterial activity of small-molecule quaternary ammonium salts is regulated by the long hydrophobic chain, which could affect the plasma membrane [18, 19]. Consequently, steps (3) and (4) could be enhanced by increasing the length of the substituted alkyl chain (quaternary ammonium group). It is possible that a polyprenol with a long hydrophobic chain and several isopentyl groups, such as a quaternary ammonium salt derivative, could bind more readily to the plasma membrane, ultimately increasing damage.

### Cytotoxic and genotoxic assay

**Fig. 4:**
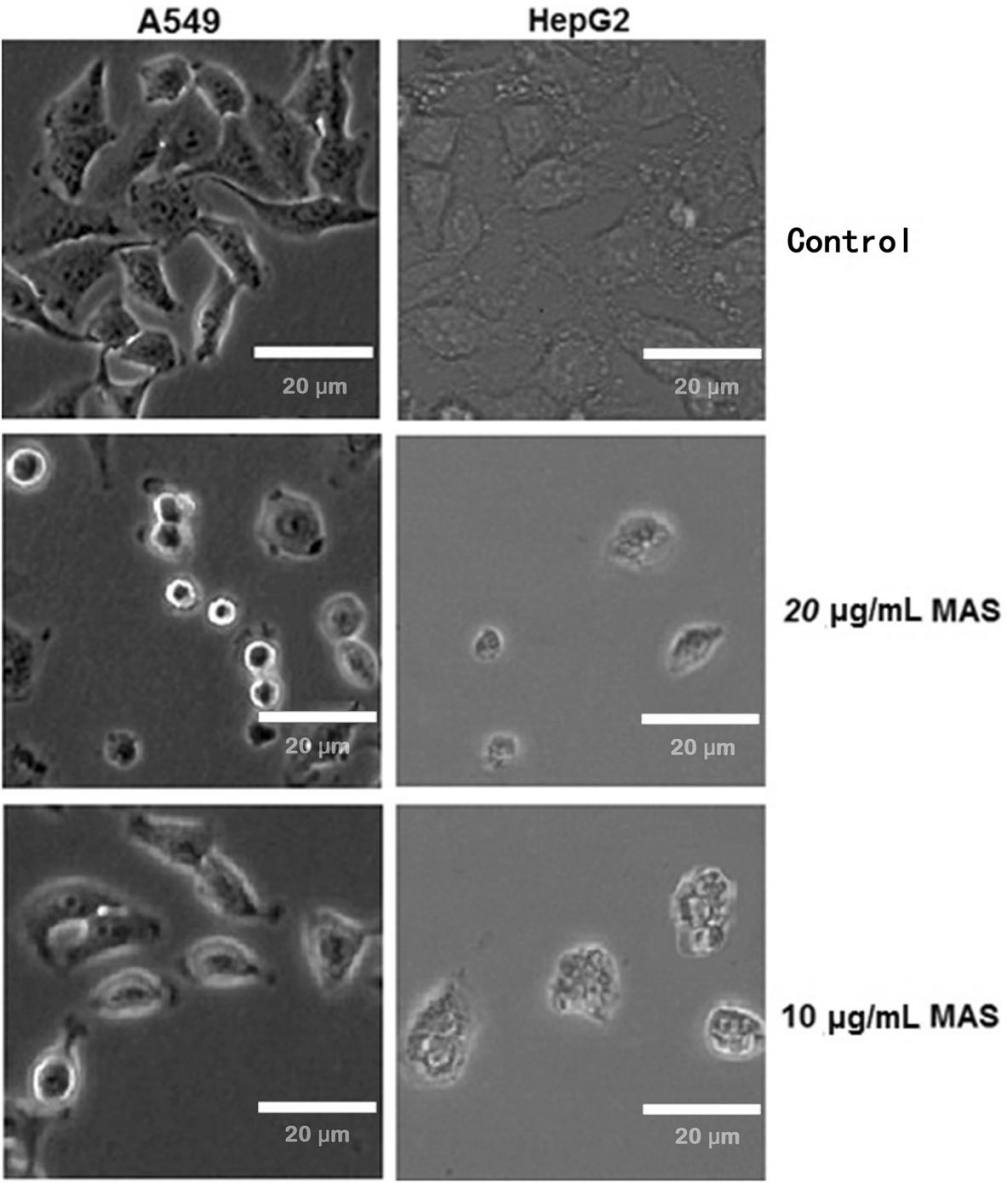
Microscopic images of A549 and HepG2 cells treated with MAS

**Fig. 5:**
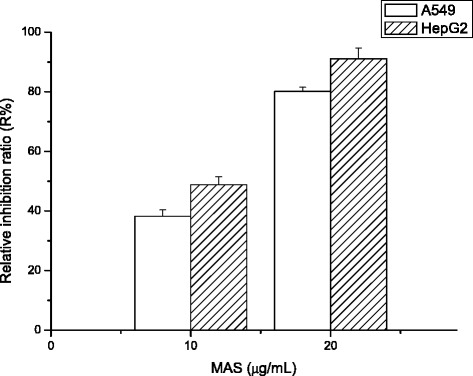
Cell viability effects of MAS at different concentration in A549 and HepG2 cells using the MTT assay (Mean ± SD, *n* = 3)

Cytotoxicity toward two cell lines (A549 and HepG2) was evaluated by MTT assay. The results (Table 3) showed that both GAS and MAS exhibited cytotoxic activity toward both cell lines, whereas GTF and MTF exhibited cytotoxic activity toward only the HepG2 cell line (IC_50_ of 33.1–39.8 μg/mL). The other compounds were found to be inactive because their IC_50_ values were greater than 50 μg/mL. In addition, MAS showed the highest activity toward HepG2 cells, with an IC_50_ of 10.1 μg/mL. GAS and MAS both exhibited stronger cytotoxic activity toward HepG2 (IC_50_ of 10.1–11.6 μg/mL) compared with A549 (IC_50_ of 13.8–13.9 μg/mL) cells. These results indicate that the quaternary polyprenyl ammonium salt derivatives (GAS and MAS) have higher cytotoxic activity (A549 and HepG2 cells) than the other nitrogenated and haloid polyprenol derivatives tested. Moreover, the polyprenyl derivatives of the same type but with different numbers of carbons (GAS with MAS, GTF with MTF) exhibited significantly different cytotoxic activities toward HepG2 cells. Subsequently, the highest activity MAS treated tumor cells were fixed and then examined by microscopy, with the results shown in Fig. 4. In cells untreated with derivative (control) and the cells were well spread out. In contrast, the MAS treated cells showed pronounced shrinkage with much apoptotic debris. MAS caused more serious structural damage to both the A549 and HepG2 cells with the increase of concentration of MAS. Relative inhibition ratio (R%) of 20 μg/mL MAS treating A549 and HepG2 were 80.1 % and 91.1 %, respectively (Fig. 5). By contrast, R% of 10 μg/mL MAS treating A549 and HepG2 were 38.2 % and 48.8 %, respectively.

**Table 3:** Cytotoxic activity of different samples

Samples	IC_50_ (μg/mL) ± SEM, *n* = 3	
	A549	HepG2
GPH	>50	>50
MPH	>50	>50
GAM	>50	>50
MAM	>50	>50
GAS	13.9 ± 0.2 a	11.6 ± 0.3 a
MAS	13.8 ± 0.2 a	10.1 ± 0.3 b
GTF	>50	39.8 ± 0.3 c
MTF	>50	33.1 ± 0.3 d
GCH	>50	>50
MCH	>50	>50
EP	0.38	0.22

The same lowercase letters in the column of the same group indicate no statistical difference (Tukey’s HSD test, *p* > 0.05). When IC_50_ > 50 μg/mL denotes inactive cytotoxic activity. *EP* etoposide

Genotoxic study of A549 and HepG2 treated with MAS was evaluated by comet assay. The results obtained in the comet assay showed that the DNA damage in A549 and HepG2 cells exposed to all 10, 20 and 40 μg/mL MAS was statistically significant in comparison to the control (Table 4). The induced genotoxicity in both A549 and HepG2 cells followed a direct dose–response effect with positive induction at 10, 20 and 40 μg/mL doses of MAS for both the percentage of DNA in the tail (% DNA tail) and tail moment (arbitrary units).

**Table 4:** The genotoxic effects of MAS at different concentration in A549 and HepG2 cells using the comet assay (Mean ± SEM, *n* = 2)

Compound, concentration (μg/mL)	% DNA tail		Tail moment (arbitrary units)	
	A549	HepG2	A549	HepG2
MAS				
10	31.03 ± 3.31*	35.58 ± 2.84*	34.73 ± 3.02*	36.78 ± 2.98*
20	57.99 ± 4.02*	59.92 ± 4.14*	54.87 ± 4.77*	57.45 ± 4.63*
40	69.49 ± 4.89*	73.95 ± 5.23*	69.06 ± 5.11*	74.21 ± 3.96*
Control	9.15 ± 1.21	9.49 ± 1.10	4.95 ± 0.95	5.12 ± 0.87
EP (0.5 μg/mL)	70.08 ± 4.33*	77.12 ± 4.01*	75.54 ± 3.86*	80.63 ± 4.55*

^a^Mean ± standard error from 2 independent experiments; 100 cells were counted for each experiment

*P < 0.01 versus control using Student’s t-test

Many studies have shown that quaternary ammonium salt derivatives have higher efficiency against tumor cells compared with the original compounds. Giraud [20] reported that quaternary ammonium salt derivatives conjugated with the melphalan alkylating agent showed micromolar-range antiproliferative activity against Saos-2 human osteosarcoma and HEMCSS human chondrosarcoma cells based on their ability to combine with proteoglycans. Gil’s study [21] further suggested that introducing cationic quaternary ammonium onto β-cyclodextrin to confer nanoparticles with non-negative zeta potential values increased the ability of the nanoparticles to penetrate the endothelial cell monolayer of bovine brain microvessels. The quaternary ammonium groups of cationic lipids could be used for transfection of various biologically active compounds (e.g., hormones, peptides, nucleosides, polynucleotides) into cells of both animal and plant origin [22]. Research on the antitumor activity of cationic lipids revealed that the presence of a quaternary ammonium group is crucial for various biological activities, and there is growing interest in chemical and biological studies of polyprenols and corresponding derivatives related to the study of posttranslational protein prenylation. Accordingly, the low toxicity of polyprenols when cultured with susceptible cells prompted us to investigate possible ways in which the synthesis of polyprenyl derivatives with positive charges can be used in immunology [23]. Research on the synthesis of secondary and tertiary polyprenylamines has been reported [24], and these compounds showed higher antitumor and antiviral activities compared with the original compounds [25, 26].

## Conclusions

In this study, nitrogenated and haloid derivatives of C_50_–C_60_ and C_70_–C_120_ polyprenols were synthesized, and the antibacterial activities against *E. coli* and *S. aureus*, cytotoxic and genotoxic activities against A549 and HepG2 cells were investigated. Overall, the quaternary polyprenyl ammonium salt derivatives GAS and MAS exhibited higher antibacterial (*E. coli* and *S. aureus*) and cytotoxic activity (A549 and HepG2 cells) compared with the other polyprenol derivatives evaluated. The DNA damage in HepG2 cells suggests that MAS induced A549 and HepG2 cells death via apoptotic pathway. This study provides new evidence supporting the medical use of polyprenol derivatives against bacterial and tumor diseases.

## Methods

### Materials

Dried GBL and MBL were collected in October 2014 from China’s Jiangsu Province. The plants were identified and authenticated by Prof. Cheng-Zhang Wang at the Institute of Chemical Industry of Forestry Products, CAF, China. Standard polyprenols (C_50_, C_55_, C_60_, C_75_–C_105_, C_110_, C_115_, C_120_) were purchased from Larodan Fine Chemical Co., Ltd, (Malmo, Sweden). Gentamycin sulfate (Sigma G3632, 100 mg) and the emulsifiers Span 80 (Sigma, 85548, 500 mL) and Tween 80 (Sigma, P1754, 500 mL) were purchased from Sigma-Aldrich Co., Ltd, (Shanghai, China). Two types of bacteria (*Escherichia coli* NCTC 12923 and *Staphylococcus aureus* ATCC 25923) were obtained from the China Center for Type Culture Collection (CCTCC, Wuhan, China). Two cell lines (A549, a human lung carcinoma cell line, CCL-185, and HepG2, a human hepatocellular liver carcinoma cell line, HB-8065) were purchased from the American Type Culture Collection (ATCC, USA). Reagents for cell culture and assays were of analytical grade, as follows: HEPES (*N*-2-hydroxyethylpiperazine-*N*-2-ethanesulfonic acid), L-glutamine, penicillin, streptomycin, sodium pyruvate, glucose and 3-(4,5-dimethylthiazol-2-yl)-2,5-diphenyltetrazolium bromide (Sigma, USA); RPMI-1640 (Roswell Park Memorial Institute medium, Gibco and Hyclone laboratories, USA); Ham’s/F12 (Nutrient mixture F-12), DMEM (Dulbecco’s Modified Eagle’s Medium) and FBS (fetal bovine serum, Hyclone Laboratories, USA). A Bruker AV-300 high-performance digital NMR spectrometer, TMS internal standard, Thermo Scientific Multiskan GO enzyme-labeled instrument (200–1000 nm), and Merck HPTLC plate, Silica gel 60 were all used in this study.

### Extraction, isolation and purification

GBL and MBL air-dried in the shade were pulverized (1 kg each) and extracted three times with a total of 3 L of absolute alcohol for 12 h at 75 °C; the samples were concentrated to yield extracts (220 g from GBL; 185 g from MBL), which were then mixed with 1 L 50 % NaOH-water solution for 2 h at 85 °C. The hydrolysates were extracted three times with 1 L of petroleum ether (b.p. 90–120 °C). The solvents were evaporated under vacuum to yield total non-saponifiable lipid extracts (42 g from GBL; 25 g from MBL), which were each dissolved in a solvent mixture (acetone : methanol = 85:15, v/v) for a solid : liquid ratio of 1:6–1:8 (g/mL) and then stored for 2 h at −20 °C. The dissolved material was concentrated to yield products in the form of a red oil (from GBL) and a brown oil (from MBL). The red and brown oils were fractionated by molecular distillation at a feed temperature of 60 °C, distillation temperature of 280 °C, feed flow rate of 180 mL/h, scraper rate of 300 rpm, and operating pressure of 0.1–0.5 Pa to yield heavy distillates in the form of a dark-red oil (26 g from GBL) and a dark-brown oil (14 g from MBL). GBP and MBP were further purified from a portion of the heavy distillates by flash column chromatography (Merck, Kiesel gel 60; 0.063–0.2 mm particle size; 4 × 60 cm) using petroleum ether (b.p. 60–90 °C) (5 × 150 mL) and 1 %, 2 %, 3 %, and 4%ethyl ether/petroleum ether (5 × 250 mL) as eluents. GBP (1.3 g) and MBP (1.1 g) were obtained from the petroleum/ethyl ether (97 %:3 %, v/v, GBP; 96 %:4 %, v/v, MBP) portion [1].

### Synthesis methods

#### GPH and MPH

Diethyl azodicarboxylate (0.14 g, 0.80 mmol) was added with stirring at 30 °C (Ar) to a solution of GBP (0.81 g, 0.70 mmol) or MBP (0.55 g, 0.68 mmol), phthalimide (0.13 g, 0.88 mmol), and Ph_3_P (0.24 g, 0.92 mmol) in 5 mL of THF. The reaction mixture was kept for 2 h at 30 °C and concentrated to dryness *in vacuo*. The residue was triturated with ether, and the insoluble material was filtered out. The filtrate was concentrated *in vacuo*, and the residue (1.0 g from GBP; 0.8 g from MBP) was chromatographed on 35 g of SiO_2_ in ether-hexane (1:4, v/v) to yield 0.59 g (68 %) of GPH and 0.45 g (70 %) of MPH as the same light-yellow oil with an Rf of 0.5–0.55 (ether-hexane, 1:4).

#### GAM and MAM

A solution of GPH (0.38 g, 0.32 mmol) or MPH (0.30 g, 0.33 mmol) and N_2_H_4_•H_2_O (0.18 g, 3.6 mmol) in a mixture of THF (5 mL) and MeOH (2 mL) was heated for 3 h at 50 °C (Ar) and diluted with ether. The solution was washed with a saturated solution of NaHCO_3_ and water. The organic layer was dried with Na_2_SO_4_, and the solvent was evaporated *in vacuo*. The residue (0.31 g from GBP; 0.25 g from MBP) was chromatographed on 30 g of Al_2_O_3_. A chloroform-methanol gradient elution (up to 5 % methanol) yielded 0.18 g (54 %) of GAM and 0.13 g (53 %) of MAM as the same light-yellow oil with an Rf of 0.65–0.68 (CHCl_3_: MeOH:H_2_O, 45:15:1, v/v).

#### GAS and MAS

Triethylamine (0.13 g, 1.3 mmol) was added to a solution of GBP (0.27 g, 0.23 mmol) or MBP (0.18 g, 0.22 mmol) in dichloromethane (5 mL). One hundred microliters of a 10 % solution of POCl_3_ (20 *μ*L, 0.22 mmol) in dichloromethane was added to the reaction mixture with stirring at −60 °C, and the solution was stirred at −60 °C for 2 h. A mixture of THF-H_2_O (1.05 mL, 20:1) was then added, followed by stirring for 2 h. The solvents were evaporated to dryness. Heptane (10 mL) was added to the residue, and the solution was kept at −20 °C over night. The solution was then filtered through a PTFE filter (0.22 *μ*m, Thermo Fisher). The combined heptane solutions were concentrated and dissolved into the upper layer of the equilibrium mixture of n-butanol-water (5 mL), and the solution was washed with the lower layer of the same equilibrium mixture (3 × 1 mL). The upper layer was concentrated, and heptane (1 mL) was added to the residue; the mixture was then extracted with acetonitrile (6 × 2 mL). The combined acetonitrile extracts were concentrated to yield the desired products: a light-yellow oil containing 0.21 g (75 %) of GAS and 0.15 g (73 %) of MAS, with an Rf of 0.5–0.55 (CHCl_3_: MeOH:H_2_O, 40:10:1, v/v).

#### GTF and MTF

Trifluoroacetic anhydride (0.11 g, 0.52 mmol) was added to a solution of GBP (0.55 g, 0.48 mmol) or MBP (0.38 g, 0.48 mmol) in dichloromethane (10 mL), and the reaction mixture was stirred at 0 °C for 2 h. The solution was washed with a saturated solution of NaHCO_3_ and water to neutrality. The organic layer was dried with Na_2_SO_4_, and the solvent was evaporated *in vacuo* and concentrated to yield the desired products: a yellow oil containing 0.51 g (93 %) of GTF and 0.36 g (95 %) of MAS, with an Rf of 0.75–0.80 (ethyl acetate : petroleum ether, 10:90, v/v).

#### GCH and MCH

Chloroacetic anhydride (0.08 g, 0.47 mmol) was added to a solution of GBP (0.53 g, 0.45 mmol) or MBP (0.36 g, 0.45 mmol) in dichloromethane (10 mL), and the reaction mixture was stirred at room temperature for 2 h. The solution was washed with a saturated solution of NaHCO_3_ and water to neutrality. The organic layer was dried with Na_2_SO_4_, and the solvent was evaporated *in vacuo* and concentrated to yield the desired products: a yellow oilcontaining0.48 g (91 %) of GCH and 0.33 g (92 %) of MCH, with an Rf of 0.70–0.75 (ethyl acetate: petroleum ether, 10:90, v/v).

### ^1^H NMR data

**GPH**: ^1^H NMR (300 MHz, CDCl_3_ δ_H_); 7.65–7.88 (m, Ph-H), 5.29 (br-t, =CH-CH_2_-N), 5.15 (m,=CH), 4.28 (br-d, N-CH_2_), 1.93–2.17 (m,-CH_2_), 1.73 (br-s, α-*cis*-CH_3_), 1.69 (br-s, *cis*-CH_3_), 1.61 (br-s, *trans*-CH_3_).

**MPH**: ^1^H NMR (300 MHz, CDCl_3_ δ_H_); 7.67–7.87 (m, Ph-H), 5.30 (br-t, =CH-CH_2_-N), 5.14 (m, =CH), 4.29 (br-d, N-CH_2_), 1.95–2.20 (m, −CH_2_), 1.72 (br-s, α-*cis*-CH_3_), 1.70 (br-s, *cis*-CH_3_), 1.61 (br-s, *trans* -CH_3_).

**GAM**: ^1^H NMR (300 MHz, CDCl_3_ δ_H_); 5.28 (br-t, =CH-CH_2_-N), 5.13 (m, =CH), 3.26 (br-d, N-CH_2_), 1.90–2.15 (m, −CH_2_), 1.73 (br-s, α-*cis*-CH_3_), 1.71 (br-s, *cis*-CH_3_), 1.60 (br-s, *trans*-CH_3_).

**MAM**: ^1^H NMR (300 MHz, CDCl_3_ δ_H_); 5.29 (br-t, =CH-CH_2_-N), 5.14 (m, =CH), 3.24 (br-d, N-CH_2_), 1.92–2.17 (m, −CH_2_), 1.72 (br-s, α-*cis*-CH_3_), 1.70 (br-s, *cis*-CH_3_), 1.61 (br-s, *trans*-CH_3_).

**GAS**: ^1^H NMR (300 MHz, CDCl_3_:CD_3_OD = 4:1 δ_H_); 5.12 (m, =CH-CH_2_-N), 4.96 (m, =CH), 3.43 (br-d, *trans*-CH_2_-N), 3.41 (br-d, *cis*-CH_2_-N), 3.13 (m, CH_3_-CH_2_-N), 1.84–2.01 (m, −CH_2_), 1.75 (br-s, α-*cis*-CH_3_), 1.55 (br-s, *cis*-CH_3_), 1.49 (br-s, *trans*-CH_3_), 1.48 (br-s, *trans*-(CH_3_)_2_C = CH), 1.22 (m, CH_3_-CH_2_-N).

**MAS**: ^1^H NMR (300 MHz, CDCl_3_:CD_3_OD = 4:1 δ_H_); 5.14 (m, =CH-CH_2_-N), 4.98 (m, =CH), 3.44 (br-d, *trans*-CH_2_-N), 3.42 (br-d, *cis*-CH_2_-N), 3.15 (m, CH_3_-CH_2_-N), 1.85–2.03 (m, −CH_2_), 1.75 (br-s, α-*cis*-CH_3_), 1.54 (br-s, *cis*-CH_3_), 1.47 (br-s, *trans*-CH_3_), 1.46 (br-s, *trans*-(CH_3_)_2_C = CH), 1.21 (m, CH_3_-CH_2_-N).

**GTF**: ^1^H NMR (300 MHz, CDCl_3_ δ_H_); 5.40 (m, =CH-CH_2_-O-C = O), 5.10 (m, =CH), 4.78–4.81 (br-d, =CH-CH_2_-O-C = O), 2.00–2.02 (m, −CH_2_), 1.80 (br-s, α-*cis*-CH_3_), 1.67 (br-s, *cis*-CH_3_), 1.60 (br-s, *trans*-CH_3_), 1.25, 1.43, 1.57 (br-s, *trans*-(CH_3_)_2_C = CH).

**MTF**: ^1^H NMR (300 MHz, CDCl_3_ δ_H_); 5.41 (m, =CH-CH_2_-O-C = O), 5.12 (m, =CH), 4.80–4.83 (br-d, =CH-CH_2_-O-C = O), 2.01–2.04 (m, −CH_2_), 1.79 (br-s, α-*cis*-CH_3_), 1.68 (br-s, *cis*-CH_3_), 1.60 (br-s, *trans*-CH_3_), 1.25, 1.43, 1.57 (br-s, *trans*-(CH_3_)_2_C = CH).

**GCH**: ^1^H NMR (300 MHz, CDCl_3_ δ_H_); 5.38 (m, =CH-CH_2_-O-C = O), 5.13 (m, =CH), 4.66–4.68 (br-d, =CH-CH_2_-O-C = O), 1.99–2.11 (m, −CH_2_), 1.77 (br-s, α-*cis*-CH_3_), 1.68 (br-s, *cis*-CH_3_), 1.60 (br-s, *trans*-CH_3_), 1.26, 1.45, 1.59 (br-s, *trans*-(CH_3_)_2_C = CH).

**MCH**: ^1^H NMR (300 MHz, CDCl_3_ δ_H_); 5.39 (m, =CH-CH_2_-O-C = O), 5.14 (m, =CH), 4.68–4.70 (br-d, =CH-CH_2_-O-C = O), 2.00–2.12 (m, −CH_2_), 1.75 (br-s, α-*cis*-CH_3_), 1.70 (br-s, *cis*-CH_3_), 1.60 (br-s, *trans*-CH_3_), 1.26, 1.44, 1.58 (br-s, *trans*-(CH_3_)_2_C = CH).

### Determination of antibacterial activity

*Escherichia coli* and *Staphylococcus aureus* antibacterial tests were carried out using Oxford cup assays [27]. All ten polyprenol derivatives (GPH, MPH, GAM, MAM, GAS, MAS, GTF, MTF, GCH and MCH), GBP and MBP were diluted to 500 μg/mL, and the resulting solutions were added to Oxford cups, which were placed at equal distances on the agar surface. The zone of inhibition for each concentration was measured after 24 h of incubation at 37 °C. The same procedure was repeated in triplicate. Gentamycin sulfate (GS) was used as a positive control, and the test samples were prepared as a microemulsion (Span 80 : Tween 80 = 0.514:0.486, wt/wt, HLB value = 9.5) using the inverse phase emulsification (EIP) method. A blank microemulsion (only water with the emulsifiers) was used as the negative control. After 24 h of incubation, the diameter of the inhibition halos was observed (measured in mm including disc size). All tests were performed in triplicate, and the observed values of the inhibition halos are expressed as mean values with standard error of the mean (SEM).

### Determination of the minimum inhibitory concentration (MIC)

The MIC values were determined using the broth dilution method at seven sample concentrations obtained via serial dilution (250, 125, 62.5, 31.3, 15.6, 7.8, 3.9 μg/mL). Solutions showing no visible cell growth were subcultured and incubated at 37 °C.

### Time-killing curves of *E. coli* and *S. aureus* treated with GAS and MAS at different concentration

Time-killing curve [28] studies for *E. coli* and *S. aureus* were conducted in duplicate on separate days using GAS and MAS at different concentration (0.5, 1 and 2 times the MIC). An overnight culture of the isolate was diluted 30-fold with prewarmed Ca-MHB and incubated at 37 °C until it reached late-log-phase growth. The bacterial suspension was diluted with Ca-MHB based on the absorbance at 630 nm; the concentration of the bacterial suspension in each flask was approximately 10^9^ CFU/mL. The experiment was conducted for 24 h in a shaker water bath set at 37 °C. A sample was taken every 2 h for the first 12 h, every 6 h from 12 to 24 h, and every 12 h from 24 to 48 h. Total bacterial populations were quantified by spiral plating 10-fold serial dilutions of the samples (50 μL) onto MHA plates. The plates were incubated in a humidified incubator (37 °C) for 18 to 24 h, and the bacterial density for each sample was determined using a CASBA-4 colony scanner and software.

### Cell culture and treatment of MAS

The human epithelial cell line A549 was grown in Ham’s/F12 medium containing 2 mmol/L *L*-glutamine supplemented with 100 U/mL penicillin, streptomycin and 10 % fetal bovine serum at 5 % CO_2_ and 37 °C. The human hepatic cell line HepG2 was grown in Dulbecco’s modified Eagle’s medium (DMEM). Test samples were prepared as microemulsions (Span 80: Tween 80 = 0.514:0.486, wt/wt, HLB value = 9.5) using the inverse-phase emulsification (EIP) method. A blank microemulsion (only water with emulsifiers) served as the negative control. The assays were performed in triplicate using etoposide (EP) as the reference drug. All samples, controls and solutions were filter sterilized (sterile 0.22 *μ*m filter, Millex®).

### Cell viability assay

Cell viability (MTT assay) toward the two cell lines (A549 and HepG2) was evaluated using a modified method [29]. In brief, cell lines were suspended in RPMI-1640 containing 10 % FBS, plated in a 96-well plate at 1 × 10^4^cells (100 μL) per well, and incubated at 37 °C in a humidified atmosphere (95 % air, 5 % CO_2_). After 24 h, an additional aliquot of medium (100 μL) containing the test compound and vehicle was added to a final concentration of 50 g/mL with 0.2 % DMSO and further incubated for 3 days. The cells were subsequently fixed with 95 % EtOH, stained with crystal violet solution and lysed with a solution of 0.1 mol/L HCl in MeOH, after which the absorbance was measured at 550 nm. The A549 and HepG2 cells were stained with MTT (3-(4,5-dimethylthiazol-2-yl)-2,5-diphenyl tetrazolium bromide). IC_50_ values were determined as the concentrations of drug or sample required to achieve 50 % cell growth inhibition. To examine the morphology of derivative treated tumor cells, A549 and HepG2 cells were preseeded on the coverslip at a density of 2 × 10^4^ cells/mL for 24 h at 37 °C in a 5 % CO_2_ atm. The derivative solution was then added, with the high and low concentration, and incubated for a further 24 h. The cells were fixed with 1 % paraformaldehyde (PFA) in PBS and then treated and observed as described above. The data were expressed as the percentage of viable cells compared to the survival of the control group (untreated cells as controls of 100 % viability). Relative inhibition ratio (R%) was calculated according to formula (1). The data were expressed as the mean ± SD of three individual measurements.

1 $$ \mathrm{R}\%=\left(1-\frac{{\mathrm{Absorbance}}_{\mathrm{test}}}{{\mathrm{Absorbance}}_{\mathrm{control}}}\right)\times 100\% $$

### Comet assay

Comet assay was performed as described with some specific modifications according to previous reports (Ali et al., 2010). Approximately 7 × 10^4^ cells/well were seeded in a 12-well plate. After 24 h of seeding, cells were treated with different concentrations of MAS for 24 h. After the treatments with samples, the cells were trypsinized and re-suspended in DMEM added with 10 % FBS and the cell suspension was centrifuged at 2 500 g (1000 rpm) for 5 min at 4 °C. The cell pellet was suspended in ice-chilled phosphate buffer saline for comet assay. Thereafter, 15 μL of cell suspension (approximately 2 × 10^4^ cells) was mixed with 85 μL of 0.5 % low melting-point agarose and it was layered on the end of a plain glass slide, pre-coated with a layer of 200 μL 1 % normal agarose. Subsequently, it was covered with a third layer of 100 μL 0.5 % low melting-point agarose. Then, the slides were immersed in the lysis solution (2.5 mol/L NaCl, 0.1 mol/L Na_2_EDTA and 0.01 mol/L Tris with 1 % Triton X-100 and 10 % DMSO, pH 10) for 12 h at 4 °C. The slides were immersed into a cold electrophoresis solution (0.3 mol/L NaOH, 0.001 mol/L Na_2_EDTA and 0.2 % DMSO, pH ≥ 13) for 20 min at 4 °C. Electrophoresis was carried out at 15 V for 20 min at 4 °C. The slides were neutralized with 0.4 mol/L tris buffer (pH 7.5) and stained with ethidium bromide (20 μg/mL, 75 μL/slide). The slides were stored at 4 °C until scoring. Slides were processed for analysis at 400 × magnification using an image analysis system (Comet assay IV, Kinetic Imaging) attached to a fluorescent microscope (Nikon Eclipse E200). An undamaged cell resembles an intact nucleus without a tail while a damaged cell has the appearance of a comet. Te percentage of DNA in the tail (% DNA tail) and tail moment (arbitrary units) were measured as comet parameters. Images from 100 random cells (50 from each replicate slide) were analyzed for each experiment. All the values were obtained from the mean ± standard error (SEM) of 2 independent experiments: in total 200 cells for each concentration. The statistical analysis was performed using SPSS (version 12.0). All data were presented as arithmetic mean ± standard error. Student’s *t*-test was selected for the statistical approach and it was used to evaluate the signifcance of the difference in DNA damage between the control and treated cells. Results were considered statistically signifcant at *P* < 0.05 or *P* < 0.01.

## Abbreviations

GAS: Quaternary polyprenyl ammonium salt derivatives from the leaves of *Ginkgo biloba*
MAS: Quaternary polyprenyl ammonium salt derivatives from the leaves of *Morus alba* (MBL)
MIC: Minimum inhibitory concentration
MTT: 3-(4,5-dimethylthiazol-2-yl)-2,5-diphenyl tetrazolium bromide
